# Supplementary material for: Dimensionality in recurrent spiking networks: Global trends in activity and local origins in connectivity
Source: PLoS Comput Biol. 2019 Jul 12;15(7):e1006446. doi: 10.1371/journal.pcbi.1006446 (PMC6655892; doi:10.1371/journal.pcbi.1006446)
Supplement: S1 File — (PDF) [file pcbi.1006446.s001.pdf]

Supplementary Material – Dimensionality in recurrent spiking networks: global trends in activity and local origins in connectivity

Contents

1 Introducing the framework **1**

2 Dimensionality of the network response without inputs **2**
- 2.1 Average of $\boldsymbol{C}$ . . . 3
- 2.2 Trace of C . . . 5
- 2.3 Average and Trace of $\boldsymbol{C}^2$ . . . 7
- 2.4 Dimensionality of the response . . . 8
- 2.5 First order expansion of the dimensionality . . . 9

3 Dimensionality of the response to inputs **11**
- 3.1 Unidimensional input . . . 12
- 3.2 Extension to multidimensional cumulants and inputs . . . 13
- 3.3 Full dimensional input . . . 15

1 Introducing the framework

We consider a network of N neurons with N_E excitatory and N_I inhibitory neurons. Spike trains $s_i(t) = \sum_j \delta(t - t^i_j)$ of neurons $i = 1...N$ are modeled as realizations of Poisson processes with time-dependent rates $y_i(t)$. We have $y_i(t) = \langle s_i(t)\rangle$ where $\langle ..\rangle$ denotes the mathematical expectation across trials. Under the assumption that the process is stationary we can replace the expectation over trials with the expectation over time. Neurons fire a spike train $\boldsymbol{s}$ with a fluctuating rate vector $\boldsymbol{y}$. A spike in neuron i temporarily influences the firing rate in neuron j with a time course governed by the interaction kernel G_{ij}. The interaction kernel can be written in terms of two components: the synaptic strength W_{ij} and postsynaptic filter A_{ii} which encapsulates the time dependency: $G_{ij} = A_{ii} \cdot W_{ij}$. The evolution of the rate is then given by:

$$\boldsymbol{y}(t) = \boldsymbol{y}_0 + \int_{-\infty}^{\infty} \boldsymbol{A}(t')\boldsymbol{W}\boldsymbol{s}(t - t')\, \mathrm{d}t' = \boldsymbol{y}_0 + (\boldsymbol{A} * \boldsymbol{W}\boldsymbol{s})(t)\ , \quad (1)$$

where $\boldsymbol{y}_0$ is the baseline firing rate of all neurons. Due to the linearity of the dynamics, multiple spikes are superimposed linearly. In the presence of an external input $\boldsymbol{\xi}$ the equation above becomes:

$$\boldsymbol{y}(t) = \boldsymbol{y}_0 + \int_{-\infty}^{\infty} \boldsymbol{A}(t')\left(\boldsymbol{W}\boldsymbol{s}(t - t') + \boldsymbol{\xi}(t - t')\right)\, \mathrm{d}t'\ . \quad (2)$$

By taking the Fourier transform of this expression we obtain:

$$\boldsymbol{y} = \boldsymbol{y}_0 + \boldsymbol{A} \cdot (\boldsymbol{W}\boldsymbol{s} + \boldsymbol{\xi})\ . \quad (3)$$

By averaging over trials we have:

$$\langle \boldsymbol{y} \rangle = (\mathcal{I} - \boldsymbol{G})^{-1} \left(\langle \boldsymbol{y}_0 \rangle + \boldsymbol{A} \cdot \langle \boldsymbol{\xi} \rangle \right) = \Delta \left(\langle \boldsymbol{y}_0 \rangle + \boldsymbol{A} \cdot \langle \boldsymbol{\xi} \rangle \right) \ , \tag{4}$$

where $\mathcal{I}$ is the identity matrix and we refer to $\Delta = (\mathcal{I} - \boldsymbol{G})^{-1}$ as the propagator. The above equation is valid in the frequency domain but in general we don't distinguish in our notation between variables in time domain or frequency domain, e.g. $\boldsymbol{\xi}(t)$ and $\boldsymbol{\xi}(w)$, with the relevant variable assumed to be clear from context.

The spectral matrix $\boldsymbol{C}(w)$ is given by:

$$\begin{aligned} \boldsymbol{C} = \langle \boldsymbol{y}\boldsymbol{y}^* \rangle &= \Delta \langle \boldsymbol{y}_0 \boldsymbol{y}_0^T \rangle \Delta^* + \Delta \left(\boldsymbol{A} \langle \boldsymbol{\xi}\boldsymbol{\xi}^T \rangle \boldsymbol{A}^* \right) \Delta^* \\ &= \Delta \boldsymbol{C}_0 \Delta^* + \Delta \left(\boldsymbol{A} \boldsymbol{C}_{inp} \boldsymbol{A}^* \right) \Delta^* \ . \end{aligned} \tag{5}$$

where $\boldsymbol{C}_0$ and $\boldsymbol{C}_{inp}$ represent respectively the correlation of spontaneous activity and external input. The same equation is shown in the main text (cfr. Eq. 2).

Our paper focuses on how the *dimensionality* of the a network's response is determined by its spontaneous dynamics and input. A standard way to assess the dimensionality of the response of the network begins by looking at the activity of pairs of neurons and assessing how correlated they are. If all neurons are highly correlated then intuitively the response will be low dimensional, and if they are all completely independent then the response will be higher dimensional, but the precise relationships between pairwise correlation values and overall dimensionality can be subtle. A way to make the connection quantitative is to consider the distribution of the eigenvalues of the covariance matrix:

$$Dim(\boldsymbol{y}) = \frac{(\mathrm{Tr}\ \boldsymbol{C})^2}{\mathrm{Tr}\ \boldsymbol{C}^2} \tag{6}$$

This expression can also be rearranged to be:

$$\begin{aligned} \frac{(\mathrm{Tr}\ \boldsymbol{C})^2}{\mathrm{Tr}\ \boldsymbol{C}^2} &\approx \frac{N \langle C_{ii} \rangle^2}{\langle (C_{ii})^2 \rangle + (N-1) \langle (C_{ij})^2 \rangle} \\ &= \frac{N \langle C_{ii} \rangle^2}{\langle C_{ii} \rangle^2 + Var(C_{ii}) + (N-1) \left(\langle C_{ij} \rangle^2 + Var(C_{ij}) \right)} \ , \end{aligned} \tag{7}$$

where C_{ii} and C_{ij} denote respectively diagonal and off-diagonal elements of the correlation matrix, $\langle .. \rangle$ being their average.

In the following we will derive equations for the dimensionality of the response as a function of the connectivity matrix $\boldsymbol{W}$. We will first focus in the case without the input term ($\boldsymbol{\xi} = 0$) and then address the case with inputs.

2 Dimensionality of the network response without inputs

In this case the only contribution to the spectral matrix $\boldsymbol{C}$ is given by $\Delta \boldsymbol{C}_0 \Delta^*$, that is, the recurrent activity induced by the spontaneous spikes emitted by every single neuron. We want to express $\boldsymbol{C}$ in terms of the connectivity matrix $\boldsymbol{W}$ and its building blocks: the motifs and cumulants. Toward this end we rewrite the propagator as an expansion:

$$\Delta = (I - \boldsymbol{G})^{-1} = \sum_{m=0}^{\infty} \boldsymbol{G}^m \ . \tag{8}$$

By writing Δ in this form we can then write $\boldsymbol{C}$ as an expansion:

$$C_{ij} = \sum_{m=0}^{\infty}\sum_{n=0}^{\infty}\sum_{k=1}^{N}(\boldsymbol{G}^m)_{ik}(\boldsymbol{C}_0)_{kk}(\boldsymbol{G}^{*n})_{kj} \ . \tag{9}$$

This expansion sums up all possible contributions of order $m+n$ to the spectral matrix. By way of assuming that cellular responses are homogeneous across the network so that $\boldsymbol{A} = g(w)\mathcal{I}$ (for simplicity with g real if not stated otherwise) and that baseline auto-correlations of each neuron are identical, $\boldsymbol{C}_0 = c_0\mathcal{I}$, the expression above can be written as:

$$\begin{aligned} C_{ij} &= \sum_{m,n=0}^{\infty}\sum_{k=1}^{N} g^m(\boldsymbol{W}^m)_{ik}c_0 g^n(\boldsymbol{W}^T)^n_{kj} = \\ &= c_0\sum_{m,n=0}^{\infty}\sum_{k=1}^{N} g^{m+n}(\boldsymbol{W}^m)_{ik}(\boldsymbol{W}^T)^n_{kj} \end{aligned} \tag{10}$$

Similarly we can write $\boldsymbol{C}^2$ as:

$$\begin{aligned} (\boldsymbol{C}^2)_{ij} &= \sum_k C_{ik}C_{kj} \\ &= \sum_{m,n=0}^{\infty}\sum_{a=1}^{N} g^m(W^m)_{ia}c_0 g^n(W^T)^n_{ak} \sum_{p,q=0}^{\infty}\sum_{b=1}^{N} g^p(W^p)_{kb}c_0 g^q(W^T)^n_{bj} \\ &= c_0^2\sum_{p,q=0}^{\infty}\sum_{m,n=0}^{\infty}\sum_{a=1}^{N}\sum_{b=1}^{N} g^{m+n+p+q}(W^m)_{ia}(W^T)^n_{ak}(W^p)_{kb}(W^T)^q_{bj} \ . \end{aligned} \tag{11}$$

We will start by calculating the average and trace of $\boldsymbol{C}$.

2.1 Average of $\boldsymbol{C}$

The average covariance can be written as:

$$\begin{aligned} \langle \boldsymbol{C} \rangle &= \frac{c_0}{N^2}\sum_{i,j=1}^{N}\sum_{m,n=0}^{\infty}\sum_k g^{m+n}W^m_{ik}(W^{Tn})_{kj} = \\ &= \frac{c_0}{N^3}\sum_{m,n=0}^{\infty}(Ng)^{m+n}\mu_{m,n} \ . \end{aligned} \tag{12}$$

where we defined $\mu_{m,n} = \sum_{k,i,j=1}^{N} W^m_{ik}(W^T_{kj})^n/N^{m+n-1} = \langle \boldsymbol{W}^m\boldsymbol{W}^{Tn}\rangle/N^{m+n-3}$ to be the empirical probability of observing an (m,n) motif in the network. We now proceed by expressing $\langle \boldsymbol{W}^m\boldsymbol{W}^{Tn}\rangle$ as a function of different motifs in the network. This approach was developed and performed in [17] and we simply follow it here, including the details as they will be relevant to our further calculations. We will use the following notation:

$$\begin{aligned} &\boldsymbol{u} = (1,1..,1)^T/\sqrt{N} \ , \\ &\boldsymbol{H} = \boldsymbol{u}\boldsymbol{u}^T \ , \\ &\boldsymbol{\Theta} = \boldsymbol{I} - \boldsymbol{H} \ , \\ &\boldsymbol{W}^{\theta}_n = (\boldsymbol{W}\Theta)^{n-1}\boldsymbol{W} \ . \end{aligned} \tag{13}$$

We can then write $\langle \boldsymbol{W}^m \boldsymbol{W^T}^n \rangle$ as:

$$
\begin{aligned}
N\langle \boldsymbol{W}^m \boldsymbol{W^T}^n \rangle &= \boldsymbol{u}^T \boldsymbol{W}^m \boldsymbol{W^T}^n \boldsymbol{u} \\
&= \boldsymbol{u}^T \left(\underbrace{\boldsymbol{W}(\boldsymbol{\Theta}+\boldsymbol{H})\boldsymbol{W}(\boldsymbol{\Theta}+\boldsymbol{H})...\boldsymbol{W}}_{\text{n factors of W}}(\boldsymbol{\Theta}+\boldsymbol{H}) \underbrace{\boldsymbol{W}^T(\boldsymbol{\Theta}+\boldsymbol{H})\boldsymbol{W}^T(\boldsymbol{\Theta}+\boldsymbol{H})...\boldsymbol{W}^T}_{\text{m factors of } \boldsymbol{W}^T} \right) \boldsymbol{u} \\
&= \sum_{\substack{\{n_1,...,n_t\}\in\mathcal{C}(n) \\ \{m_1,...,m_s\}\in\mathcal{C}(m)}} \boldsymbol{u}^T \left(\boldsymbol{W}^{\theta}_{n_t} \boldsymbol{H} \boldsymbol{W}^{\theta}_{n_{t-1}} \boldsymbol{H} ... \boldsymbol{W}^{\theta}_{n_1} (\boldsymbol{\Theta}+\boldsymbol{H}) \boldsymbol{W}^{T\theta}_{m_1} \boldsymbol{H} \boldsymbol{W}^{T\theta}_{m_2} \boldsymbol{H} ... \boldsymbol{W}^{T\theta}_{m_s} \right) \boldsymbol{u} \\
&= \sum_{\substack{\{n_1,...,n_t\}\in\mathcal{C}(n) \\ \{m_1,...,m_s\}\in\mathcal{C}(m)}} \boldsymbol{u}^T \boldsymbol{W}^{\theta}_{n_t} \boldsymbol{u}\boldsymbol{u}^T \boldsymbol{W}^{\theta}_{n_{t-1}} \boldsymbol{u}\boldsymbol{u^T} ... \boldsymbol{W}^{\theta}_{n_1} (\boldsymbol{\Theta}+\boldsymbol{u}\boldsymbol{u^T}) \boldsymbol{W}^{T\theta}_{m_1} \boldsymbol{u}\boldsymbol{u^T} \boldsymbol{W}^{T\theta}_{m_2} \boldsymbol{u}\boldsymbol{u^T} ... \boldsymbol{W}^{T\theta}_{m}
\end{aligned}
\tag{14}
$$

where $\mathcal{C}(n)$ is the set of ordered partitions of n: each element of $\mathcal{C}(n)$ is an ordered set of numbers, e.g. $\{n_1, ..., n_t\}$, that sum up to n.

Now we introduce the following definitions to simplify Eq. 14:

$$
\begin{aligned}
\kappa_n =& \frac{1}{N^{n+1}} \sum_{i,j} (\underbrace{\boldsymbol{W}\boldsymbol{\Theta}\boldsymbol{W}...\boldsymbol{\Theta}\boldsymbol{W}}_{\text{n factors of } \boldsymbol{W}})_{ij} = \frac{1}{N^n} \boldsymbol{u}^T [(\boldsymbol{W}\boldsymbol{\Theta})^{n-1}\boldsymbol{W}] \boldsymbol{u} = \frac{1}{N^n} \boldsymbol{u}^T \boldsymbol{W}^{\theta}_n \boldsymbol{u} \ , \\
\kappa^{\text{div}}_{n,m} =& \frac{1}{N^{n+m+1}} \sum_{i,j} \left(\underbrace{\boldsymbol{W}\boldsymbol{\Theta}\boldsymbol{W}...\boldsymbol{\Theta}\boldsymbol{W}}_{\text{n factors of } \boldsymbol{W}} \boldsymbol{\Theta} \underbrace{\boldsymbol{W}^T\boldsymbol{\Theta}\boldsymbol{W}^T...\boldsymbol{\Theta}\boldsymbol{W}^T}_{\text{m factors of } \boldsymbol{W}^T} \right)_{ij} \\
=& \frac{1}{N^{n+m}} \boldsymbol{u}^T \left((\boldsymbol{W}\boldsymbol{\Theta})^{n-1} \boldsymbol{W}\boldsymbol{\Theta}\boldsymbol{W}^T (\boldsymbol{\Theta}\boldsymbol{W}^T)^{m-1} \right) \boldsymbol{u} = \frac{1}{N^{n+m}} \boldsymbol{u}^T \boldsymbol{W}^{\theta}_n \boldsymbol{\Theta} \boldsymbol{W}^{\theta T}_m \boldsymbol{u} \ ,
\end{aligned}
\tag{15}
$$

Following [17] we will refer to κ_n as an n-chain cumulant while we call $\kappa^{div}_{n,m}$ a cumulant for an (n,m)-divergent motif. These cumulants determine the part of the probability of having an n-chain or an (n,m)-divergent motif that cannot be predicted by lower order statistics, i.e. cumulants of lower order. We can rewrite Eq. 14 as:

$$
\begin{aligned}
N\langle \boldsymbol{W}^m \boldsymbol{W^T}^n \rangle =& \sum_{\substack{\{n_1,...,n_t\}\in\mathcal{C}(n) \\ \{m_1,...,m_s\}\in\mathcal{C}(m)}} N^{n+m} (\kappa_{n_t}\kappa_{n_{t-1}}...\kappa_{n_2})(\kappa^{div}_{m_1+n_1} + \kappa_{n_1+m_1})(\kappa_{m_2}...\kappa_{m_s}) \\
=& \sum_{\substack{\{n_1,...,n_t\}\in\mathcal{C}(n) \\ \{m_1,...,m_s\}\in\mathcal{C}(m)}} N^{n+m} \left(\prod_{i=2}^{t} \kappa_{n_i} \right) (\kappa^{div}_{n_1,m_1} + \kappa_{n_1}\kappa_{m_1}) \left(\prod_{i=2}^{s} \kappa_{m_j} \right) + \kappa^{div}_{n,m} \ ,
\end{aligned}
\tag{16}
$$

where, in evaluating these terms, we use the convention $(\prod_{i=2}^{t} \kappa_{n_i}) = 1$ if $t = 1$.

Now we can plug this result in Eq. 12:

$$
\begin{aligned}
\langle \boldsymbol{C} \rangle =& \frac{c_0}{N^3} \sum_{m,n=0}^{\infty} (Ng)^{m+n} \mu_{m,n} \\
=& \frac{c_0}{N} \sum_{m,n=0}^{\infty} (Ng)^{m+n} \left(\sum_{\substack{\{n_1,...,n_t\}\in\mathcal{C}(n) \\ \{m_1,...,m_s\}\in\mathcal{C}(m)}} \left(\prod_{i=2}^{t} \kappa_{n_i} \right) (\kappa^{div}_{n_1,m_1} + \kappa_{n_1}\kappa_{m_1}) \left(\prod_{i=2}^{s} \kappa_{m_j} \right) + \kappa^{div}_{n,m} \right)
\end{aligned}
\tag{17}
$$

We use the following identity:

$$\sum_{n,m=1}^{\infty} \sum_{\substack{\{n_1,...,n_t\}\in\mathcal{C}(n)\\ \{m_1,...,m_s\}\in\mathcal{C}(m)}} \left[\left(\prod_{p=1}^{t} x_{n_p}\right) z_{n_p m_q} \left(\prod_{q=1}^{s} y_{m_q}\right)\right] = \left[\sum_{i=0}^{\infty}\left(\sum_{n=1}^{\infty} x_n\right)^i\right]\left(\sum_{n,m=1}^{\infty} z_{nm}\right)\left[\sum_{j=0}^{\infty}\left(\sum_{m=1}^{\infty} y_m\right)^j\right] \tag{18}$$

to obtain the final result for the average correlation of $\boldsymbol{C}$ in terms of the cumulants statistics:

$$\langle \boldsymbol{C} \rangle = \frac{c_0}{N}\left(1-\sum_{n=1}^{\infty}(Ng)^n \kappa_n\right)^{-2}\left(1+\sum_{n,m=1}^{\infty}(Ng)^{n+m}\kappa_{n,m}^{div}\right) . \tag{19}$$

This formula shows that the average correlation depends only on the statistics of chains and divergent motifs and, as already mentioned, was derived in previous work [17]. We included here its derivation for the sake of writing a self-contained introduction to the use of cumulants, and to facilitate the use of allied methods below.

2.2 Trace of C

A similar calculation to the one above leads to the value of the trace of $\boldsymbol{C}$ as a function of the statistics of connectivity expressed in terms of the cumulants [43].

$$\begin{aligned} Tr(\boldsymbol{C}) &= c_0 \sum_{m,n=0}^{\infty} g^{m+n} Tr\left(\boldsymbol{W}^m \boldsymbol{W}^{Tn}\right) \\ &= \frac{c_0}{N}\sum_{m,n=0}^{\infty}(Ng)^{m+n}\mu_{m,n}^{Tr} . \end{aligned} \tag{20}$$

Similarly to Eq. 14 we have:

$$\begin{aligned} &Tr(\boldsymbol{W}^m\boldsymbol{W}^{Tn}) = \\ &= Tr\left(\underbrace{\boldsymbol{W}(\boldsymbol{\Theta}+\boldsymbol{H})\boldsymbol{W}(\boldsymbol{\Theta}+\boldsymbol{H})...\boldsymbol{W}}_{\text{n factors of W}}(\boldsymbol{\Theta}+\boldsymbol{H})\underbrace{\boldsymbol{W}^T(\boldsymbol{\Theta}+\boldsymbol{H})\boldsymbol{W}^T(\boldsymbol{\Theta}+\boldsymbol{H})...\boldsymbol{W}^T(\boldsymbol{\Theta}+\boldsymbol{H})}_{\text{m factors of }\boldsymbol{W}^T}\right) \\ &= \sum_{\substack{\{n_1,...,n_t\}\in\mathcal{C}(n)\\ \{m_1,...,m_s\}\in\mathcal{C}(m)}} Tr\left(\boldsymbol{W}_{n_t}^{\theta}\boldsymbol{H}\boldsymbol{W}_{n_{t-1}}^{\theta}\boldsymbol{H}...\boldsymbol{W}_{n_1}^{\theta}(\boldsymbol{\Theta}+\boldsymbol{H})\boldsymbol{W}_{m_1}^{T\theta}\boldsymbol{H}\boldsymbol{W}_{m_2}^{T\theta}\boldsymbol{H}...\boldsymbol{W}_{m_s}^{T\theta}(\boldsymbol{\Theta}+\boldsymbol{H})\right) \\ &= \sum_{\substack{\{n_1,...,n_t\}\in\mathcal{C}(n)\\ \{m_1,...,m_s\}\in\mathcal{C}(m)}} Tr\left(\boldsymbol{W}_{n_t}^{\theta}\boldsymbol{u}\boldsymbol{u}^T\boldsymbol{W}_{n_{t-1}}^{\theta}\boldsymbol{u}\boldsymbol{u}^{\boldsymbol{T}}...\boldsymbol{W}_{n_1}^{\theta}(\boldsymbol{\Theta}+\boldsymbol{u}\boldsymbol{u}^{\boldsymbol{T}})\boldsymbol{W}_{m_1}^{T\theta}\boldsymbol{u}\boldsymbol{u}^{\boldsymbol{T}}...\boldsymbol{W}_{m_s}^{T\theta}(\boldsymbol{\Theta}+\boldsymbol{u}\boldsymbol{u}^{\boldsymbol{T}})\right) \end{aligned} \tag{21}$$

Now we notice that if at least one of $\boldsymbol{H}$ is present in the product above (e.g. $s > 1$), then we can exploit the cyclical property of the trace $Tr(\boldsymbol{ABC}) = Tr(\boldsymbol{CAB}) = Tr(\boldsymbol{BCA})$ to obtain for each of these terms:

$$\begin{aligned} &Tr\left(\boldsymbol{W}_{n_t}^{\theta}...\boldsymbol{W}_{n_1}^{\theta}(\boldsymbol{\Theta}+\boldsymbol{u}\boldsymbol{u}^{\boldsymbol{T}})\boldsymbol{W}_{m_1}^{T\theta}\boldsymbol{u}\boldsymbol{u}^{\boldsymbol{T}}...\boldsymbol{W}_{m_s}^{T\theta}(\boldsymbol{\Theta}+\boldsymbol{u}\boldsymbol{u}^{\boldsymbol{T}})\right) \\ &\quad = Tr\left(\boldsymbol{u}^{\boldsymbol{T}}...\boldsymbol{W}_{m_s}^{T\theta}(\boldsymbol{\Theta}+\boldsymbol{u}\boldsymbol{u}^{\boldsymbol{T}})\boldsymbol{W}_{n_t}^{\theta}...\boldsymbol{W}_{n_1}^{\theta}(\boldsymbol{\Theta}+\boldsymbol{u}\boldsymbol{u}^{\boldsymbol{T}})\boldsymbol{W}_{m_1}^{T\theta}\boldsymbol{u}\right) \\ &\quad = \boldsymbol{u}^{\boldsymbol{T}}...\boldsymbol{W}_{m_s}^{T\theta}(\boldsymbol{\Theta}+\boldsymbol{u}\boldsymbol{u}^{\boldsymbol{T}})\boldsymbol{W}_{n_t}^{\theta}...\boldsymbol{W}_{n_1}^{\theta}(\boldsymbol{\Theta}+\boldsymbol{u}\boldsymbol{u}^{\boldsymbol{T}})\boldsymbol{W}_{m_1}^{T\theta}\boldsymbol{u} . \end{aligned} \tag{22}$$

In this equation the trace operation has become trivial as the product inside the trace is a scalar. To account for all possible terms of the form in Eq. 22 we introduce two new cumulants:

$$\begin{aligned}
\kappa^{\text{conv}}_{n,m} =& \frac{1}{N^{n+m+1}} \sum_{i,j} \Bigg(\underbrace{\boldsymbol{W}^T \boldsymbol{\Theta} \boldsymbol{W}^T ... \boldsymbol{\Theta} \boldsymbol{W}^T}_{\text{n factors of } \boldsymbol{W}^T} \boldsymbol{\Theta} \underbrace{\boldsymbol{W} \boldsymbol{\Theta} \boldsymbol{W} ... \boldsymbol{\Theta} \boldsymbol{W}}_{\text{m factors of } \boldsymbol{W}} \Bigg)_{ij} \\
=& \frac{1}{N^{n+m}} \boldsymbol{u}^T [(\boldsymbol{W}^T \boldsymbol{\Theta})^{n-1} \boldsymbol{W}^T \boldsymbol{\Theta} \boldsymbol{W} (\boldsymbol{\Theta} \boldsymbol{W})^{m-1}] \boldsymbol{u} = \frac{1}{N^{n+m}} \boldsymbol{u}^T \boldsymbol{W}^{\theta T}_n \boldsymbol{\Theta} \boldsymbol{W}^{\theta}_m \boldsymbol{u} \ , \\
\kappa^{\text{Tr}}_{n,m} =& \frac{1}{N^{n+m+1}} \sum_{i} \Bigg(\underbrace{\boldsymbol{W} \boldsymbol{\Theta} \boldsymbol{W} ... \boldsymbol{\Theta} \boldsymbol{W}}_{\text{n factors of } \boldsymbol{W}} \boldsymbol{\Theta} \underbrace{\boldsymbol{W}^T \boldsymbol{\Theta} \boldsymbol{W}^T ... \boldsymbol{\Theta} \boldsymbol{W}^T \boldsymbol{\Theta}}_{\text{m factors of } \boldsymbol{W}^T} \Bigg)_{ii} \\
=& \frac{1}{N^{n+m+1}} \text{Tr} \left[(\boldsymbol{W} \boldsymbol{\Theta})^{n-1} \boldsymbol{W} \boldsymbol{\Theta} \boldsymbol{W}^T (\boldsymbol{\Theta} \boldsymbol{W}^T)^{m-1} \boldsymbol{\Theta} \right] = \frac{1}{N^{n+m+1}} \text{Tr} \left[\boldsymbol{W}^{\theta}_n \boldsymbol{\Theta} \boldsymbol{W}^{\theta T}_m \boldsymbol{\Theta} \right] \ .
\end{aligned} \tag{23}$$

These cumulants, together with the ones defined in Eq. 15, allow us to write each term of the form highlighted by Eq. 22 as:

$$\begin{aligned}
&\boldsymbol{u}^{\boldsymbol{T}} ... \boldsymbol{W}^{T\theta}_{m_s} (\boldsymbol{\Theta} + \boldsymbol{u}\boldsymbol{u}^{\boldsymbol{T}}) \boldsymbol{W}^{\theta}_{n_t} ... \boldsymbol{W}^{\theta}_{n_1} (\boldsymbol{\Theta} + \boldsymbol{u}\boldsymbol{u}^{\boldsymbol{T}}) \boldsymbol{W}^{T\theta}_{m_1} \boldsymbol{u} \\
&= N^{n+m} \left(\prod_{i=2}^{s-1} \kappa_{m_j} \right) (\kappa^{\text{conv}}_{n_t,m_s} + \kappa_{n_t} \kappa_{m_s}) \left(\prod_{i=2}^{t-1} \kappa_{n_i} \right) (\kappa^{\text{div}}_{n_1,m_1} + \kappa_{n_1} \kappa_{m_1}) \ .
\end{aligned} \tag{24}$$

The only term that cannot be written in such a way is the one that doesn't "contain" any $\boldsymbol{H}$, which corresponds to $\kappa^{Tr}_{m,n}$. So we obtain:

$$\begin{aligned}
\text{Tr}(\boldsymbol{W}^m \boldsymbol{W}^{\boldsymbol{T}^n}) = N^{n+m} \sum_{\substack{\{n_1,...,n_t\} \in \mathcal{C}(n) \\ \{m_1,...,m_s\} \in \mathcal{C}(m)}} \left(\prod_{i=2}^{s-1} \kappa_{m_j} \right) (\kappa^{\text{conv}}_{n_t,m_s} + \kappa_{n_t} \kappa_{m_s}) \left(\prod_{i=2}^{t-1} \kappa_{n_i} \right) \\
(\kappa^{\text{div}}_{n_1,m_1} + \kappa_{n_1} \kappa_{m_1}) + N^{n+m} N \kappa^{Tr}_{n,m} \ .
\end{aligned} \tag{25}$$

We can now resum $\text{Tr}(\boldsymbol{C})$ in a similar way to how we proceeded in Eq. 17, 19 by exploiting the identity in Eq. 18. The final expression for the trace of $\boldsymbol{C}$ then becomes:

$$\begin{aligned}
\text{Tr}(\boldsymbol{C}) =& c_0 \sum_{n,m=0}^{\infty} (gN)^{n+m} \mu^{\text{Tr}}_{n,m} \\
=& c_0 \sum_{n,m=0}^{\infty} (gN)^{n+m} \sum_{\substack{\{n_1,...,n_t\} \in \mathcal{C}(n) \\ \{m_1,...,m_s\} \in \mathcal{C}(m)}} \left(\prod_{i=2}^{s-1} \kappa_{m_j} \right) (\kappa^{\text{conv}}_{n_t,m_s} + \kappa_{n_t} \kappa_{m_s}) \left(\prod_{i=2}^{t-1} \kappa_{n_i} \right) \\
& (\kappa^{\text{div}}_{n_1,m_1} + \kappa_{n_1} \kappa_{m_1}) + N \kappa^{Tr}_{n,m} \\
=& c_0 \left(1 - \sum_{n=1}^{\infty} (gN)^n k_n \right)^{-2} \left(1 + \sum_{n,m=1}^{\infty} (gN)^{n+m} k^{div}_{n,m} \right) \left(1 + \sum_{n.m=1}^{\infty} (gN)^{n+m} k^{conv}_{n,m} \right) \\
& + c_0 N \sum_{n,m=1}^{\infty} (gN)^{n+m} \kappa^{Tr}_{n,m}
\end{aligned} \tag{26}$$

This expression defines the value of $Tr(C)$ as a function of the statistics of the connectivity W.

In order to calculate the dimensionality of the response, besides the term $Tr(\boldsymbol{C})$, we also need the term $Tr(\boldsymbol{C}^2)$.

2.3 Average and Trace of $\boldsymbol{C}^2$

We show how a similar calculation leads to the value of the average and trace of $\boldsymbol{C^2}$ as a function of the statistics of connectivity expressed in terms of the cumulants.

$$
\begin{aligned}
\langle \boldsymbol{C}^2 \rangle &= c_0^2 \sum_{m,n,p,q=0}^{\infty} g^{m+n+p+q} \langle \boldsymbol{W}^m \boldsymbol{W}^{Tn} \boldsymbol{W}^p \boldsymbol{W}^{Tq} \rangle \\
&= \frac{c_0^2}{N^3} \sum_{m,n,p,q=0}^{\infty} (Ng)^{m+n+p+q} \mu_{m,n,p,q} \ . \\
Tr(\boldsymbol{C}^2) &= c_0^2 \sum_{m,n,p,q=0}^{\infty} g^{m+n+p+q} Tr \left(\boldsymbol{W}^m \boldsymbol{W}^{Tn} \boldsymbol{W}^p \boldsymbol{W}^{Tq} \right) \\
&= \frac{c_0^2}{N} \sum_{m,n,p,q=0}^{\infty} (gN)^{m+n+p+q} \mu^{Tr}_{m,n,p,q} \ .
\end{aligned} \tag{27}
$$

This sum, that runs over four indices, can be interpreted in the same way as the ones above. To handle all the possible terms in this sum we would have to introduce new cumulants that account for more and more complicated motifs. For example, one of these cumulants would be

$$
\kappa^{div,conv,div}_{n,m,p} = \frac{1}{N^{n+m+p}} \boldsymbol{u}^T \boldsymbol{W}^{\theta T}_n \boldsymbol{\Theta} \boldsymbol{W}^{\theta}_m \boldsymbol{\Theta} \boldsymbol{W}^{\theta T}_p \boldsymbol{u} \ . \tag{28}
$$

Similarly we would have to use $\kappa^{conv,div}_{m,n,p}$, $\kappa^{div,conv}_{m,n,p}$, $\kappa^{conv,div,conv}_{m,n,p}$, $\kappa^{div,conv,div}_{m,n,p}$. Although it is possible to define these terms and calculate their contribution to $\mathrm{Tr}(\boldsymbol{C}^2)$ and $\langle \boldsymbol{C}^2 \rangle$, we will assume in the following derivation that they are zero. This assumption corresponds to assuming that only terms that are constructed from 2nd order contributions are nonzero. This corresponds to terms of the following form in the case of $\mathrm{Tr}(\boldsymbol{C}^2)$:

$$
\begin{aligned}
&\boldsymbol{u^T} ... \boldsymbol{W}^{T\theta}_{q_o} (\boldsymbol{\Theta} + \boldsymbol{u}\boldsymbol{u^T}) \boldsymbol{W}^{\theta}_{n_t} ... \boldsymbol{W}^{\theta}_{n_1} (\boldsymbol{\Theta} + \boldsymbol{u}\boldsymbol{u^T}) \boldsymbol{W}^{T\theta}_{m_1} .. \\
&\qquad .. \boldsymbol{W}^{T\theta}_{m_s} (\boldsymbol{\Theta} + \boldsymbol{u}\boldsymbol{u^T}) \boldsymbol{W}^{\theta}_{p_r} ... \boldsymbol{W}^{\theta}_{p_1} (\boldsymbol{\Theta} + \boldsymbol{u}\boldsymbol{u^T}) \boldsymbol{W}^{T\theta}_{q_1} \boldsymbol{u} \\
&= N^{n+m+p+q} \left(\prod_{i=2}^{o-1} \kappa_{q_i} \right) (\kappa^{\mathrm{conv}}_{q_o,n_t} + \kappa_{n_t} \kappa_{q_o}) \left(\prod_{i=2}^{t-1} \kappa_{n_i} \right) (\kappa^{\mathrm{div}}_{n_1,m_1} + \kappa_{n_1} \kappa_{m_1}) .. \\
&\qquad .. \left(\prod_{i=2}^{s-1} \kappa_{m_j} \right) (\kappa^{\mathrm{conv}}_{m_s,p_r} + \kappa_{m_s} \kappa_{p_r}) \left(\prod_{i=2}^{r-1} \kappa_{p_i} \right) (\kappa^{\mathrm{div}}_{p_1,q_1} + \kappa_{p_1} \kappa_{q_1}) \ .
\end{aligned} \tag{29}
$$

A similar term would appear in the expansion of $\langle \boldsymbol{C}^2 \rangle$, except for the lack of the convergent motifs represented above by $\kappa^{\mathrm{conv}}_{q_o,n_t}$.

The only other terms that are not in this form are the trace terms corresponding to $\kappa^{\mathrm{Tr}} = \kappa^{conv,div,conv,div}_{m,n,p,q}$. The order of these terms is given by $m+n+p+q$. A second order approximation keeps account of all terms such that $m+n+p+q \leq 2$. Thus the

final expression for $\mathrm{Tr}(\boldsymbol{C}^2)$ is:

$$\begin{aligned}
\mathrm{Tr}(\boldsymbol{C}^2) = &\frac{c_0^2}{N}\sum_{n,m=0}^{\infty}(gN)^{n+m}\mu^{\mathrm{Tr}}_{n,m,p,q}\\
=&c_0^2\sum_{n,m,p,q=0}^{\infty}(gN)^{n+m}\sum_{\substack{\{n_1,\ldots,n_t\}\in\mathcal{C}(n)\\ \{m_1,\ldots,m_s\}\in\mathcal{C}(m)\\ \{p_1,\ldots,p_r\}\in\mathcal{C}(p)\\ \{q_1,\ldots,q_o\}\in\mathcal{C}(q)}}(gN)^{n+m+p+q}\left(\prod_{i=2}^{o-1}\kappa_{q_i}\right)\left(\kappa^{\mathrm{conv}}_{q_o,n_t}+\kappa_{n_t}\kappa_{q_o}\right)\left(\prod_{i=2}^{t-1}\kappa_{n_i}\right)\\
&...\left(\kappa^{\mathrm{div}}_{n_1,m_1}+\kappa_{n_1}\kappa_{m_1}\right)\left(\prod_{i=2}^{s-1}\kappa_{m_j}\right)\left(\kappa^{\mathrm{conv}}_{m_s,p_r}+\kappa_{m_s}\kappa_{p_r}\right)\left(\prod_{i=2}^{r-1}\kappa_{p_i}\right)\left(\kappa^{\mathrm{div}}_{p_1,q_1}+\kappa_{p_1}\kappa_{q_1}\right)\\
&+\sum_{m,n,p,q=0}^{\infty}(Ng)^{m+n}Nc_0^2\,\mu^{Tr}_{m,n,p,q}\\
=&c_0^2\left(1-\sum_{n=1}^{\infty}(gN)^n k_n\right)^{-4}\left(1+\sum_{n,m=1}^{\infty}(gN)^{n+m}k^{div}_{n,m}\right)^2\left(1+\sum_{n.m=1}^{\infty}(gN)^{n+m}k^{conv}_{n,m}\right)^2\\
&+Nc_0^2\sum_{n,m,p,q=1}^{\infty}(gN)^{n+m+p+q}\kappa^{Tr}_{n,m,p,q}
\end{aligned} \tag{30}$$

This equation approximates $\mathrm{Tr}(\boldsymbol{C}^2)$ to second order in the cumulant expansion.

Similarly, the formula for $\langle \boldsymbol{C}^2\rangle$ is:

$$\begin{aligned}
\langle \boldsymbol{C}^2\rangle =& \frac{c_0^2}{N^3}\sum_{n,m=0}^{\infty}(gN)^{n+m}\mu_{n,m,p,q}\\
=&\frac{c_0^2}{N}\left(1-\sum_{n=1}^{\infty}(gN)^n k_n\right)^{-4}\left(1+\sum_{n,m=1}^{\infty}(gN)^{n+m}k^{div}_{n,m}\right)^2\left(1+\sum_{n.m=1}^{\infty}(gN)^{n+m}k^{conv}_{n,m}\right)\ .
\end{aligned} \tag{31}$$

2.4 Dimensionality of the response

Upon calculating $\mathrm{Tr}(\boldsymbol{C})$ and $\mathrm{Tr}(\boldsymbol{C}^2)$ we can return to the initial definition of the dimensionality of the response (Eq. 6):

$$Dim(\boldsymbol{y}) = \frac{(\mathrm{Tr}\ \boldsymbol{C})^2}{\mathrm{Tr}\ \boldsymbol{C}^2}\ . \tag{32}$$

In the case without input this definition corresponds to the ratio between Eq. 26 (squared) and Eq. 30. Rather than copying the lengthy expressions of Eq. 26, 30 this large fraction, we analyze here the interesting limit of an Erdos-Reyni network. In this limit, we will highlight and explain the structure of Eq. 6.

For an Erdos Reyni network all cumulants are zero, except for $k_1 = p$ (p is the probability that each edge is present in the graph) and the trace cumulants

$k^{Tr}_{0,0} = (1 - 1/N)$. In this limit Eq. 6 becomes:

$$\begin{aligned} Dim(\boldsymbol{y}) = \frac{(\mathrm{Tr}\ \boldsymbol{C})^2}{\mathrm{Tr}\ \boldsymbol{C}^2} &= \frac{\left(c_0\,(1 - gNk_1)^{-2} + c_0 N k^{\mathrm{Tr}}_{0,0}\right)^2}{c_0^2\,(1 - gNk_1)^{-4}\ + c_0^2 N k^{\mathrm{Tr}}_{0,0,0,0}} \\ &= \frac{\left((1 - gNp)^{-2}\ + (1 - \frac{1}{N})N\right)^2}{(1 - gNp)^{-4}\ + (1 - \frac{1}{N})N}\ . \end{aligned} \tag{33}$$

From this formula we see that when $gk_1 << \frac{1}{N}$ then $Dim(\boldsymbol{y}) \sim N$, while as $gk_1 \to \frac{1}{N}$ we obtain $Dim(\boldsymbol{y}) \to 1$. This behaviour can be described in the following way: for p small enough the structure of $\boldsymbol{C}$ is fully diagonal and all the elements are equal to c_0. In this regime all the neurons in the network act independently and contribute equally to $Dim(\boldsymbol{y})$. As p increases, the length of chains in the network become longer and longer and more and more neurons start interacting, until the main mode of the network fully dominates the dynamics, corresponding to $Dim(\boldsymbol{y}) = 1$. In Fig. 3a of the main text we see how Eq. 33 is in agreement with the full value of the dimension.

2.5 First order expansion of the dimensionality

To understand the role of different cumulants in increasing or decreasing the dimensionality of the network response we compute a first order Taylor expansion as follows. We expand the full formula for $Dim(\boldsymbol{y})$ around the Erdos Renyi solution and compute the coefficients for the chain, convergent, divergent and trace cumulants. The general form of the dimensionality formula is:

$$Dim(\boldsymbol{y}) = \frac{(\mathrm{Tr}\ \boldsymbol{C})^2}{\mathrm{Tr}\ \boldsymbol{C}^2}\ . \tag{34}$$

So the derivative around the Erdos-Renyi solution for a given cumulant k will be:

$$\partial_k\Big|_{ER} dim(\boldsymbol{y}) = \partial_k\Big|_{ER} \frac{(\mathrm{Tr}\ \boldsymbol{C})^2}{\mathrm{Tr}\ \boldsymbol{C}^2}\ . \tag{35}$$

where the subscript ER indicates the Erdos-Renyi solution for a given p (that we will specify in the following). We will temporarily use ∂_k in place of $\partial_k\Big|_{ER}$ wherever useful for conciseness.

$$\begin{aligned} \partial_k\Big|_{ER} Dim(\boldsymbol{y}) &= \frac{\partial_k(\mathrm{Tr}\ \boldsymbol{C})^2 \mathrm{Tr}(\boldsymbol{C}^2) - (\mathrm{Tr}\ \boldsymbol{C})^2 \partial_k(\mathrm{Tr}\boldsymbol{C}^2)}{(\mathrm{Tr}\ \boldsymbol{C}^2)^2} = \\ &= \frac{2\partial_k(\mathrm{Tr}\ \boldsymbol{C})\mathrm{Tr}(\boldsymbol{C}^2) - (\mathrm{Tr}\ \boldsymbol{C})^2 \partial_k(\mathrm{Tr}\boldsymbol{C}^2)}{(\mathrm{Tr}\ \boldsymbol{C}^2)^2} \end{aligned} \tag{36}$$

We treat separately the two partial differentiations $\partial_k(\mathrm{Tr}\ \boldsymbol{C})$ and $\partial_k(\mathrm{Tr}\ \boldsymbol{C}^2)$. As we are interested in understanding the overall influence of motifs on the dimensionality, we differentiate with respect to the total cumulants sum for each kind of motif. In other

words, we define:

$$
\begin{aligned}
k^{ch} &= \sum_{n=1}^{\infty}(gN)^n k_n \\
k^{div} &= \sum_{n,m=1}^{\infty}(gN)^{n+m} k^{div}_{n,m} \\
k^{conv} &= \sum_{n,m=1}^{\infty}(gN)^{n+m} k^{conv}_{n,m} \\
k^{\mathrm{Tr}}_2 &= \sum_{n,m=1}^{\infty}(gN)^{n+m} \kappa^{Tr}_{n,m} \\
k^{\mathrm{Tr}}_4 &= \sum_{n,m,p,q=1}^{\infty}(gN)^{n+m} \kappa^{Tr}_{n,m,p,q}
\end{aligned}
\tag{37}
$$

and we also assume N to be big enough so that $N-1 \approx N$. We define:

$$
r = (1 - gNk_1)^{-1} \ . \tag{38}
$$

The first differentation is, according to Eq. 26:

$$
\begin{aligned}
&\partial_{k^{ch}}(\mathrm{Tr}\ \boldsymbol{C}) = \\
&\quad = \partial_{k^{ch}}\left(c_0\left(1-\sum_{n=1}^{\infty}(gN)^n k_n\right)^{-2}\left(1+\sum_{n,m=1}^{\infty}(gN)^{n+m}k^{div}_{n,m}\right)\left(1+\sum_{n,m=1}^{\infty}(gN)^{n+m}k^{conv}_{n,m}\right)\right. \\
&\qquad\qquad \left. + c_0 N \sum_{n,m=1}^{\infty}(gN)^{n+m}\kappa^{Tr}_{n,m}\right) = \\
&\quad = \partial_{k^{ch}}\Big|_{ER}\left(c_0\left(1-k^{ch}\right)^{-2}\left(1+k^{div}\right)\left(1+k^{conv}\right)+c_0 N \kappa^{Tr}_2\right) = \\
&\quad = \left(2c_0\left(1-k^{ch}\right)^{-2}\left(1+k^{div}\right)\left(1+k^{conv}\right)+c_0 N \kappa^{Tr}_2\right)\Big|_{ER} = 2c_0\left(1-gNk_1\right)^{-3} = 2c_0 r^3 \\
&\partial_{k^{div}}(\mathrm{Tr}\ \boldsymbol{C}) = c_0\left(1-gNk_1\right)^{-2} = c_0 r^2 \\
&\partial_{k^{conv}}(\mathrm{Tr}\ \boldsymbol{C}) = c_0\left(1-gNk_1\right)^{-2} = c_0 r^2 \\
&\partial_{k^{\mathrm{Tr}}}(\mathrm{Tr}\ \boldsymbol{C}) = c_0 N \ .
\end{aligned}
\tag{39}
$$

Similarly we can compute the derivative of $\partial_k(\mathrm{Tr}\ \boldsymbol{C}^2)$ according to Eq. 30

$$
\begin{aligned}
&\partial_{k^{ch}}(\mathrm{Tr}\ \boldsymbol{C}^2) = 4c_0^2\left(1-gNk_1\right)^{-5} = 4c_0^2 r^5 \\
&\partial_{k^{div}}(\mathrm{Tr}\ \boldsymbol{C}^2) = 2c_0^2\left(1-gNk_1\right)^{-4} = 2c_0^2 r^4 \\
&\partial_{k^{conv}}(\mathrm{Tr}\ \boldsymbol{C}^2) = 2c_0^2\left(1-gNk_1\right)^{-4} = 2c_0^2 r^4 \\
&\partial_{k^{\mathrm{Tr}}}(\mathrm{Tr}\ \boldsymbol{C}^2) = c_0^2 N \ .
\end{aligned}
\tag{40}
$$

Now we combine Eq. 39 and 40 as in Eq. 36 by using:

$$
\begin{aligned}
(\mathrm{Tr}\ \boldsymbol{C})\Big|_{ER} &= c_0\left(1-gNk_1\right)^{-2} + c_0(N-1) = c_0(r^2+N-1) \sim c_0(r^2+N) \\
\left(\mathrm{Tr}\ \boldsymbol{C}^2\right)\Big|_{ER} &= c_0^2\left(1-gNk_1\right)^{-4} + c_0^2(N-1) = c_0^2(r^4+N-1) \sim c_0^2(r^4+N).
\end{aligned}
\tag{41}
$$

We obtain:

$$
\begin{aligned}
&\partial_{k^{ch}}\Big|_{ER} Dim(\boldsymbol{y}) = \\
&\quad = \frac{2(\text{Tr } \boldsymbol{C})\partial_{k^{ch}}(\text{Tr } \boldsymbol{C})\text{Tr}(\boldsymbol{C}^2) - (\text{Tr } \boldsymbol{C})^2 \partial_{k^n}(\text{Tr}\boldsymbol{C}^2)}{(\text{Tr } \boldsymbol{C}^2)^2} = \\
&= \frac{4r^3(r^2+N)}{r^4+N} - \frac{(r^2+N)^2(4r^5)}{(r^4+N)^2} \\
&\partial_{k^{div}}\Big|_{ER} Dim(\boldsymbol{y}) = \frac{2r^2(r^2+N)}{r^4+N} - \frac{(r^2+N)^2(2r^4)}{(r^4+N)^2} \\
&\partial_{k^{conv}}\Big|_{ER} Dim(\boldsymbol{y}) = \frac{2r^2(r^2+N)}{r^4+N} - \frac{(r^2+N)^2(2r^4)}{(r^4+N)^2} \\
&\partial_{k_2^{\text{Tr}}}\Big|_{ER} Dim(\boldsymbol{y}) = \frac{2N(r^2+N)}{r^4+N} \\
&\partial_{k_4^{\text{Tr}}}\Big|_{ER} Dim(\boldsymbol{y}) = -\frac{(r^2+N)^2 N}{(r^4+N)^2}
\end{aligned}
\tag{42}
$$

These partial derivatives can be regarded as the coefficients for the Taylor expansion around the Erdos Renyi case. Evaluating the sign of these coefficients shows the following interesting result: increasing values for all the cumulants except for one of the trace cumulants tend to reduce the dimensionality of the network response.

We close by noting an important caveat to the above derivation. The cumulants $k^{\text{Tr}}_{n,m}$ and $k^{\text{Tr}}_{n,m,p,q}$ are strictly related by their internal combinatorics: the same terms that build up the first also contribute to the second but to a different extent. As different building blocks contribute differently there is no straightforward relation between the two. In fact, at lower orders the two can be strongly positively correlated, as shown in Fig. 3f of the main text.

3 Dimensionality of the response to inputs

In Eq. 5 we pointed out two different contributions to the covariance matrix of the network response. The first is the covariance of the intrinsic activity (i.e. the covariance induced by the propagation of the baseline activity of each neuron), the second is the covariance induced by external inputs. In the previous section we extensively analysed the first term $\boldsymbol{C}_{int} = \Delta \boldsymbol{C}_0 \Delta^*$. We will refer to this term as internal correlation, as it is the contribution to the total correlation of the internally generated spontaneous activity. We now analyze the second term $\boldsymbol{C}_{ext} = \Delta(\boldsymbol{A}\boldsymbol{C}_{inp}\boldsymbol{A}^*)\Delta^*$, where $\boldsymbol{C}_{inp} = \langle \boldsymbol{\xi}\boldsymbol{\xi}^T \rangle$ is the covariance of the inputs being fed to the network.

We remind readers that that the formula for the dimensionality, Eq. 6, is quadratic in the trace of $\boldsymbol{C}$, which in turn means that it accounts for interactions (mixed terms) between inputs and the spontaneous activity. Eq. 6 reads:

$$
Dim(\boldsymbol{y}) = \frac{(\text{Tr } \boldsymbol{C}_{int} + \text{Tr } \boldsymbol{C}_{ext})^2}{\text{Tr } (\boldsymbol{C}_{int} + \boldsymbol{C}_{ext})^2} = \frac{(\text{Tr } \boldsymbol{C}_{int})^2 + (\text{Tr } \boldsymbol{C}_{ext})^2 + 2(\text{Tr } \boldsymbol{C}_{ext} \cdot \text{Tr } \boldsymbol{C}_{ext})}{\text{Tr}(\boldsymbol{C}_{int}^2) + \text{Tr}(\boldsymbol{C}_{ext}^2) + \text{Tr}(2\boldsymbol{C}_{int} \cdot \boldsymbol{C}_{ext})} \tag{43}
$$

where we recall that $\boldsymbol{C}_{int}, \boldsymbol{C}_{ext}$ are symmetric and so they commute.

Next, we will begin our analysis from the case of a unidimensional input and then generalize.

3.1 Unidimensional input

In the case where the input is unidimensional, we will denote the input with $\sqrt{c_\xi}\boldsymbol{\xi}$ where c_ξ is a constant representing the intensity of the input and $\boldsymbol{\xi}$ is a unit-norm vector representing its direction. If we consider $\boldsymbol{\xi}$ to be a constant vector, then $\boldsymbol{C}_{inp} = c_\xi\langle\boldsymbol{\xi}\boldsymbol{\xi}^T\rangle = \boldsymbol{\xi}\boldsymbol{\xi}^T$. Furthermore we also consider the case where $A = g(w)\mathcal{I}$ (g for simplicity). The correlation matrix then reduces to:

$$\begin{aligned}\boldsymbol{C}_{ext} = c_\xi\Delta(\boldsymbol{A}\boldsymbol{C}_{inp}\boldsymbol{A}^*)\Delta^* &= \Delta(\boldsymbol{A}\boldsymbol{\xi}\boldsymbol{\xi}^T\boldsymbol{A}^*)\Delta^* = \Delta(g^2\boldsymbol{\xi}\boldsymbol{\xi}^T)\Delta^* \\ =& c_\xi\sum_{m,n=0}^{\infty}\sum_{k=1}^{N} g^m(\boldsymbol{W}^m)_{ik}(g^2\boldsymbol{\xi}\boldsymbol{\xi}^T)g^n(\boldsymbol{W}^T)^n_{kj} \\ =& c_\xi\sum_{m,n=0}^{\infty} g^{m+n+2}\sum_{k,l=1}^{N}(\boldsymbol{W}^m)_{ik}(\boldsymbol{\xi}\boldsymbol{\xi}^T)_{kl}(\boldsymbol{W}^T)^n_{lj} \ ,\end{aligned} \tag{44}$$

To compute the motifs for the input we can use the same approach as used in Sec. S2.1 , but where we redefine $\boldsymbol{u}, \boldsymbol{H}, \boldsymbol{\Theta}, \boldsymbol{W}^\theta$ of Eq. 13 as:

$$\begin{aligned}&\boldsymbol{u} = \boldsymbol{\xi} \ , \\ &\boldsymbol{H}_{inp} = \boldsymbol{C}_{inp} \ , \\ &\boldsymbol{\Theta}_{inp} = \mathcal{I} - \boldsymbol{H}_{inp} \ , \\ &\boldsymbol{W}_n^{\theta-inp} = (\boldsymbol{W}\boldsymbol{\Theta}_{inp})^{n-1}\boldsymbol{W} \ .\end{aligned} \tag{45}$$

Now we notice that Eq. 44 leads to an expansion similar to the one for the spontaneous activity, except that the correlation matrix in between the Δ's is not full rank, and that we don't have $\boldsymbol{C}_{inp} \propto \mathcal{I} = \boldsymbol{\Theta} + \boldsymbol{H}$ but rather $\boldsymbol{C}_{inp} \propto \boldsymbol{H}$. So the theory already developed applies to this case with slight modifications that account for this difference. It is possible to define the cumulants for the input case to obey the exact same expression as before but where $\boldsymbol{u}, \boldsymbol{H}, \boldsymbol{\Theta}, \boldsymbol{W}^\theta$ are given by Eq. 45. We refer to these cumulants as $\tilde{\kappa}$.

The expressions for $\langle\boldsymbol{C}\rangle$ and $\mathrm{Tr}(\boldsymbol{C})$ directly generalize to this case with a simple modification due to the structure of $\boldsymbol{C}_{inp}$: the divergent motif, as a consequence of the term $(\boldsymbol{W}^m)(\boldsymbol{\xi}\boldsymbol{\xi}^T)(\boldsymbol{W}^T)^n$ is now absent as the structure of $\boldsymbol{C}_{inp}$ is not similar to the identity $\boldsymbol{C}_{inp} \propto \mathcal{I} = \boldsymbol{H} + \boldsymbol{\Theta}$ but rather $\boldsymbol{C}_{inp} \propto \boldsymbol{H}$. Thus we obtain that Eq. 26 becomes:

$$\mathrm{Tr}(\boldsymbol{C}_{ext}) = c_\xi\left(1 - \sum_{n=1}^{\infty}(gN)^n\tilde{\kappa}_n\right)^{-2}\left(1 + \sum_{n.m=1}^{\infty}(gN)^{n+m}\tilde{\kappa}^{conv}_{n,m}\right) \tag{46}$$

and Eq. 30 becomes:

$$\mathrm{Tr}(\boldsymbol{C}^2_{ext}) = c_\xi\left(1 - \sum_{n=1}^{\infty}(gN)^n\tilde{\kappa}_n\right)^{-4}\left(1 + \sum_{n.m=1}^{\infty}(gN)^{n+m}\tilde{\kappa}^{conv}_{n,m}\right)^2 \tag{47}$$

Through Eq. 46,47 we can now rewrite the dimensionality of the response in Eq. 43. The only missing piece for this equation is $\mathrm{Tr}(\boldsymbol{C}_{int}\cdot\boldsymbol{C}_{ext})$. To calculate this term we need to deal with the expansion:

$$\boldsymbol{C}_{int}\cdot\boldsymbol{C}_{ext} = \sum_{m,n,p,q=0}^{\infty} c_0c_\xi g^{m+n+p+q+2}\sum_{k,l=1}^{N}(\boldsymbol{W}^m)_{ik}(\boldsymbol{W}^T)^n_{kl}(\boldsymbol{W}^m)_{lp}(\boldsymbol{\xi}\boldsymbol{\xi}^T)_{pq}(\boldsymbol{W}^T)^n_{qj} \ . \tag{48}$$

Summing up these terms with the same assumptions used for $\boldsymbol{C}^2$ (of disregarding terms with three or more indices) yields:

$$\begin{aligned}\mathrm{Tr}(\boldsymbol{C}_{inp}\cdot\boldsymbol{C}_{ext}) = c_0 c_\xi g^2 \left(1-\sum_{n=1}^{\infty}(gN)^n\tilde{\kappa}_n\right)^{-4} &\left(1+\sum_{n.m=1}^{\infty}(gN)^{n+m}\tilde{\kappa}^{div}_{n,m}\right)^2 \\ &\left(1+\sum_{n.m=1}^{\infty}(gN)^{n+m}\tilde{\kappa}^{conv}_{n,m}\right)\end{aligned} \tag{49}$$

With Eq. 49, we now have all the terms in Eq. 43 and we can evaluate the dimensionality of the response to an input as a function of the network's connectivity statistics. The cumulants used in such an expansion are $\kappa_n, \kappa^{div}_{n,m}, \kappa^{con}_{n,m}, \kappa^{\mathrm{Tr}}_{n,m}$ and the input cumulants $\tilde{\kappa}_n, \tilde{\kappa}^{div}_{n,m}, \tilde{\kappa}^{con}_{n,m}$.

3.2 Extension to multidimensional cumulants and inputs

So far we have considered the case where cumulants κ and input cumulants $\tilde{\kappa}$ were defined using a single vector, either $\boldsymbol{u}$ or $\boldsymbol{\xi}$. These vectors were at the core of the definitions of Eq.13 and Eq.45. So far, they played the role of identifying a specific direction crucial for the propagation of the intrinsic activity (vector $\boldsymbol{u}$) and of the input signal (vector $\boldsymbol{\xi}$). A natural question that thus far remains unanswered is: how can this framework be generalized to multiple directions? We now show how to extend the results obtained above to this case. We emphasize the meaning of this extension:

- Intrinsic activity: in the case of the network activity (Eq.13) in the absence of input stimuli, adding a second direction lets us keep track of how the intrinsic activity propagates in the network along another direction. In an Erdos-Renyi network there is a single privileged direction, the maximum eigenvector, but in general multiple directions are necessary to account for the propagation of the intrinsic activity so that this can be described with a few cumulants. We will comment more on this in the following.
- Input: in the case of networks receiving inputs (Eq.45), introducing multiple directions for the cumulants allows us to account for a multidimensional input stimulus – a much more general case than the unidimensional input considered above.

Given a number P of orthogonal directions $\boldsymbol{u}_\mu$ (vectors of norm equal to one) with $\mu = 1..P$ we define $\boldsymbol{U}$ to be the matrix with $\boldsymbol{u}_\mu$ as μ-th column. Then we define:

$$\begin{aligned}\boldsymbol{H}_m &= \sum_{\mu=1}^{P}\boldsymbol{u}_\mu\boldsymbol{u}_\mu^T = \boldsymbol{U}\boldsymbol{U}^T\ , \\ \boldsymbol{\Theta}_m &= \mathcal{I}-\boldsymbol{H}_{inp}\ , \\ \boldsymbol{W}^m_n &= (\boldsymbol{W}\boldsymbol{\Theta}_m)^{n-1}\boldsymbol{W}\end{aligned} \tag{50}$$

so that cumulants can now be defined for each direction separately or altogether in a matrix form. In the first case we have:

$$\begin{aligned}\kappa^\mu_n &= \frac{1}{N^n}\boldsymbol{u_\mu}^T\boldsymbol{W}^\theta_n\boldsymbol{u_\mu}\ , \\ \kappa^{\mathrm{div}-\mu}_{n,m} &= \frac{1}{N^{n+m}}\boldsymbol{u_\mu}^T\boldsymbol{W}^\theta_n\boldsymbol{\Theta}\boldsymbol{W}^{\theta T}_m\boldsymbol{u_\mu}\ , \\ \kappa^{\mathrm{conv}-\mu}_{n,m} &= \frac{1}{N^{n+m}}\boldsymbol{u_\mu}^T\boldsymbol{W}^{\theta T}_n\boldsymbol{\Theta}\boldsymbol{W}^\theta_m\boldsymbol{u_\mu}\ , \\ \kappa^{\mathrm{Tr}}_{n,m} &= \frac{1}{N^{n+m+1}}\mathrm{Tr}\left[\boldsymbol{W}^\theta_n\boldsymbol{\Theta}\boldsymbol{W}^{\theta T}_m\boldsymbol{\Theta}\right]\ .\end{aligned} \tag{51}$$

The matrix formulation is instead:

$$
\begin{aligned}
\boldsymbol{K}_n =& \frac{1}{N^n}\boldsymbol{U}^T\boldsymbol{W}^\theta_n\boldsymbol{U} \ , \\
\boldsymbol{K}^{\mathrm{div}}_{n,m} =& \frac{1}{N^{n+m}}\boldsymbol{U}^T\boldsymbol{W}^\theta_n\boldsymbol{\Theta}\boldsymbol{W}^{\theta T}_m\boldsymbol{U} \ , \\
\boldsymbol{K}^{\mathrm{conv}}_{n,m} =& \frac{1}{N^{n+m}}\boldsymbol{U}^T\boldsymbol{W}^{\theta T}_n\boldsymbol{\Theta}\boldsymbol{W}^{\theta}_m\boldsymbol{U} \ , \\
\kappa^{\mathrm{Tr}}_{n,m} =& \frac{1}{N^{n+m+1}}\mathrm{Tr}\left[\boldsymbol{W}^\theta_n\boldsymbol{\Theta}\boldsymbol{W}^{\theta T}_m\boldsymbol{\Theta}\right] \ ,
\end{aligned}
\tag{52}
$$

where we notice that $\kappa^{\mathrm{Tr}}_{n,m}$ is a scalar by definition and is the same as in Eq. 51, and that all the matrices are diagonal as follows from the vectors $\boldsymbol{u}_\mu$ orthogonal.

These definitions can be further broadened to the case where we consider left and right matrices: $\boldsymbol{U}$ and $\boldsymbol{V}$ of the same size $N \times P$ and having orthogonal unitary vectors as columns. This definition allows us to exploit the SVD decomposition of the matrix $\boldsymbol{W}$ or $\boldsymbol{C}$ and in general we have:

$$
\begin{aligned}
&\boldsymbol{H}_m = \boldsymbol{U}\boldsymbol{V}^T \ , \\
&\boldsymbol{\Theta}_m = \mathcal{I} - \boldsymbol{H}_{inp} \ , \\
&\boldsymbol{W}^m_n = (\boldsymbol{W}\boldsymbol{\Theta}_m)^{n-1}\boldsymbol{W} \ ,
\end{aligned}
\tag{53}
$$

with cumulants:

$$
\begin{aligned}
\boldsymbol{K}_n =& \frac{1}{N^n}\boldsymbol{U}^T\boldsymbol{W}^\theta_n\boldsymbol{V} \ , \\
\boldsymbol{K}^{\mathrm{div}}_{n,m} =& \frac{1}{N^{n+m}}\boldsymbol{U}^T\boldsymbol{W}^\theta_n\boldsymbol{\Theta}\boldsymbol{W}^{\theta T}_m\boldsymbol{V} \ , \\
\boldsymbol{K}^{\mathrm{conv}}_{n,m} =& \frac{1}{N^{n+m}}\boldsymbol{U}^T\boldsymbol{W}^{\theta T}_n\boldsymbol{\Theta}\boldsymbol{W}^{\theta}_m\boldsymbol{V} \ , \\
\kappa^{\mathrm{Tr}}_{n,m} =& \frac{1}{N^{n+m+1}}\mathrm{Tr}\left[\boldsymbol{W}^\theta_n\boldsymbol{\Theta}\boldsymbol{W}^{\theta T}_m\boldsymbol{\Theta}\right] \ .
\end{aligned}
\tag{54}
$$

These definitions provide a generalized framework to exploit cumulants in multiple directions. To fully use this framework, we need to redefine Eq. 18 for the matrix case. As shown in reference 18 of the main article Eq. 18 generalizes to matrix form:

$$
\begin{aligned}
&\sum_{n,m=1}^{\infty} \sum_{\substack{\{n_1,\ldots,n_t\}\in\mathcal{C}(n)\\ \{m_1,\ldots,m_s\}\in\mathcal{C}(m)}} \left[\left(\prod_{p=1}^{t}\boldsymbol{X}_{n_p}\right)\boldsymbol{Z}_{n_p m_q}\left(\prod_{q=1}^{s}\boldsymbol{Y}_{m_q}\right)\right] \\
&= \left[\sum_{i=0}^{\infty}\left(\sum_{n=1}^{\infty}\boldsymbol{X}_n\right)^i\right]\left(\sum_{n,m=1}^{\infty}\boldsymbol{Z}_{nm}\right)\left[\sum_{j=0}^{\infty}\left(\sum_{m=1}^{\infty}\boldsymbol{Y}_m\right)^j\right]
\end{aligned}
\tag{55}
$$

where $\boldsymbol{X}_i, \boldsymbol{Y}_i, \boldsymbol{Z}_i$ are matrices for each i. Applying this identity it is possible to obtain similar expressions for all the formulas obtained up to here that involve a trace

operation. For example Eq. 26 becomes:

$$\begin{aligned}\mathrm{Tr}(\boldsymbol{C}) &= c_0 \sum_{n,m=0}^{\infty} (gN)^{n+m} \mu_{n,m}^{\mathrm{Tr}} \\ &= \mathrm{Tr}\left(c_0 \left(1 - \sum_{n=1}^{\infty} (gN)^n \boldsymbol{K}_n\right)^{-2} \left(1 + \sum_{n,m=1}^{\infty} (gN)^{n+m} \boldsymbol{K}_{n,m}^{div}\right)\right. \\ &\qquad \left.\left(1 + \sum_{n.m=1}^{\infty} (gN)^{n+m} \boldsymbol{K}_{n,m}^{conv}\right)\right) + c_0 N \sum_{n,m=1}^{\infty} (gN)^{n+m} \kappa_{n,m}^{Tr}\end{aligned} \tag{56}$$

This framework does not directly apply to Eq. 19 and other average correlation measures. The reason is that the direction that these measures involve, which allows averaging, is $\boldsymbol{u} = (1,1,1..1)^T$, and unless one of the vectors in $\boldsymbol{U}$ is this specific direction then $\langle \boldsymbol{C} \rangle$ and $\langle \boldsymbol{C}^2 \rangle$ cannot be directly expressed in terms of cumulants. To do so it is necessary to employ the machinery explained in [43]. In [43] a subpopulation approach was developed and explained in detail. Such an approach consists in choosing $\boldsymbol{U}$ where each column vector is binary (i.e., $\boldsymbol{u}_i = (1,0,0,1,0..1)^T$) and these vectors are orthogonal to one another. Such a choice constructs $\boldsymbol{u} = (1,1,1..1)^T$ by means of considering the average procedure over subpopulations. This approach can be viewed as a specific case of interest for the multidimensional cumulants covered above.

3.3 Full dimensional input

In the case of input that is of full dimensional, i.e. none of the directions of the input to the network can be neglected, then there is an alternative approach to the computation of the dimensionality. As correlation matrices are positive definite, we can write $\boldsymbol{C}_{inp} = \boldsymbol{C}_{inp}^{1/2} \boldsymbol{C}_{inp}^{1/2}$ and then rewrite $\boldsymbol{C}_{ext}$ as:

$$\begin{aligned}\boldsymbol{C}_{ext} =& \Delta(\boldsymbol{A}\boldsymbol{C}_{inp}\boldsymbol{A}^*)\Delta^* \\ =& \boldsymbol{C}_{inp}^{1/2}\boldsymbol{C}_{inp}^{-1/2}\Delta(\boldsymbol{A}\boldsymbol{C}_{inp}^{1/2}\boldsymbol{C}_{inp}^{1/2}\boldsymbol{A}^*)\Delta^*\boldsymbol{C}_{inp}^{-1/2}\boldsymbol{C}_{inp}^{1/2} \\ =& \boldsymbol{C}_{inp}^{1/2}(\mathcal{I} - \boldsymbol{C}_{inp}^{-1/2}\boldsymbol{A}\boldsymbol{W}\boldsymbol{A}\boldsymbol{C}_{inp}^{1/2})^{-1}(\mathcal{I} - \boldsymbol{C}_{inp}^{1/2}\boldsymbol{A}^*\boldsymbol{W}^*\boldsymbol{A}^*\boldsymbol{C}_{inp}^{-1/2})^{-1}\boldsymbol{C}_{inp}^{1/2} \\ =& \boldsymbol{C}_{inp}^{1/2}(\mathcal{I} - \boldsymbol{G}_{ext})^{-1}(\mathcal{I} - \boldsymbol{G}_{ext}^*)^{-1}\boldsymbol{C}_{inp}^{1/2}\end{aligned} \tag{57}$$

where $\boldsymbol{G}_{ext} = \boldsymbol{C}_{inp}^{-1/2}\boldsymbol{A}\boldsymbol{W}\boldsymbol{A}\boldsymbol{C}_{inp}^{1/2} = g^2\boldsymbol{C}_{inp}^{-1/2}\boldsymbol{W}\boldsymbol{C}_{inp}^{1/2} = g^2\boldsymbol{W}_{ext}$ is the modified effective connectivity matrix, following ideas in 18.

Although the above expression holds only in the case where the correlation matrix is full rank, we can consider that the effect of the input can be taken into account by replacing the effective connectivity matrix $\boldsymbol{G}$ with $\boldsymbol{G}_{ext}$ and accounting for the projection $\boldsymbol{C}_{inp}^{1/2}$.

The analysis that led to Eq. 26 can be adapted to account for a correlation structure

like the one in Eq. 57. In this case Eq. 20 reads:

$$
\begin{aligned}
\mathrm{Tr}(\boldsymbol{C}) &= \mathrm{Tr}(\boldsymbol{C}_{int}) + \mathrm{Tr}(\boldsymbol{C}_{ext}) \\
&= c_0 \sum_{m,n=0}^{\infty} g^{m+n} \mathrm{Tr}\left(\boldsymbol{W}^m \boldsymbol{W}^{Tn}\right) + c_{inp} \sum_{m,n=0}^{\infty} g^{2(m+n)} \mathrm{Tr}\left(\boldsymbol{C}_{inp}^{1/2} \boldsymbol{W}_{ext}^m \boldsymbol{W}_{ext}^{Tn} \boldsymbol{C}_{inp}^{1/2}\right) \\
&= c_0 \sum_{m,n=0}^{\infty} g^{m+n} \mathrm{Tr}\left(\boldsymbol{W}^m \boldsymbol{W}^{Tn}\right) + c_{inp} \sum_{m,n=0}^{\infty} g^{2(m+n)} \mathrm{Tr}\left(\boldsymbol{C}_{inp} \boldsymbol{W}_{ext}^m \boldsymbol{W}_{ext}^{Tn}\right) \\
&= \frac{c_0}{N^3} \sum_{m,n=0}^{\infty} (Ng)^{m+n} \mu_{m,n}^{\mathrm{Tr}} + \frac{c_{inp}}{N^3} \sum_{m,n=0}^{\infty} (Ng^2)^{m+n} \nu_{m,n}^{\mathrm{Tr},inp} \ .
\end{aligned}
\tag{58}
$$

To compute this formula in the cumulant framework we can use the same approach as in Sec. S3.2. The formula sheds light on the interplay between the connectivity matrix $\boldsymbol{W}$ and the input correlation $\boldsymbol{C}_{inp}$, that is expressed through $\boldsymbol{G}_{ext}$. A cumulant reduction that resums properly should take into account the eigenvectors with corresponding highest eigenvalue for all these matrices. We will not go deeper in developing such an approach for the full rank input, calculating a formula for $\nu_{m,n}^{Tr,inp}$, but here we simply stress how the effective connectivity matrix $\boldsymbol{G}_{ext}$ is defined and contributes to the dimensionality.
